# A set of shuttle plasmids for gene expression in *Acinetobacter baumannii*

**DOI:** 10.1371/journal.pone.0246918

**Published:** 2021-02-10

**Authors:** Jing Jie, Xiao Chu, Dan Li, Zhaoqing Luo

**Affiliations:** Department of Respiratory Medicine and Center of Infection and Immunity, Key Laboratory of Organ Regeneration and Transplantation of the Ministry of Education, The First Hospital of Jilin University, Changchun, China; All India Institute of Medical Sciences, Bhopal, INDIA

## Abstract

Infections caused by the emerging opportunistic bacterial pathogen *Acinetobacter baumannii* are occurring at increasingly alarming rates, and such increase in incidence is further compounded by the development of wide spread multidrug-resistant strains. Yet, our understanding of its pathogenesis and biology remains limited which can be attributed in part to the scarce of tools for molecular genetic analysis of this bacterium. Plasmids based on pWH1277 originally isolated from *Acinetobacter calcoaceticus* are the only vehicles currently available for ectopic gene expression in *Acinetobacter* species, which restricts experiments that require simultaneous analysis of multiple genes. Here, we found that plasmids of the IncQ group are able to replicate in *A*. *baumannii* and can stably co-reside with derivatives of pWH1277. Furthermore, we have constructed a series of four plasmids that allow inducible expression of Flag-tagged proteins in *A*. *baumannii* by arabinose or isopropyl β-d-1-thiogalactopyranoside. Together with constructs previously developed, these plasmids will accommodate the need in genetic analysis of this increasingly important pathogen.

## Introduction

*Acinetobacter baumannii* is a Gram-negative bacterium known for its pleomorphism and the lack of motility; it is an important opportunistic pathogen that can colonize the skin and often is isolated from the respiratory and oropharynx secretions of infected individuals [1]. Infection by *A*. *baumannii* normally occurs in immunocompromised populations and is of high incidence in hospital environments, particularly in intensive care unit (ICU) wards with chronically ill patients [2].

The high incidence of infection by *A*. *baumannii* is compounded by the dramatic increase in the development of multidrug-resistant (MDR) strains, which has generated alarms in the medical community [2, 3]. Because of the extensive antibiotic resistance spectrum associated with many of its isolates, *A*. *baumannii* is one of a group of six bacterial species that had been dubbed “ESKAPE”, which also includes *Enterococcus faecium*. *Staphylococcus aureus*, *Klebsiella pneumoniae*, *Pseudomonas aeruginosa and Enterobacter* species [4]. These bacteria not only cause the majority of nosocomial infections, but also represent distinct paradigms of pathogenesis, transmission, and resistance thus posing grave threat to hospitals [5].

The high infection incidence and resistance to multiple antibiotics have attracted considerable research attention to *A*. *baumannii* in recent years, which has generated important insights into both basic biology and pathogenesis of this pathogen [6, 7]. At least two specialized secretion systems, including a type II secretion system and a type VI secretion system have been identified in *A*. *baumannii*, which appear to contribute to its virulence, persistence and resistance [8]. Like other pathogens capable of persisting in environments of scarce nutrient, *A*. *baumannii* utilizes several proteins to effectively assimilate metal ions from the environment and host cells [9–11]. Recently, it was reported that *A*. *baumannii* modifies its cellular envelop to adapt to the environment, and in some cases such modifications also are directly linked to its resistance to antibacterial agents [12, 13]. By combination of genomic methods such as insertion sequencing (INSeq) and transposon sequencing (Tn-seq) [14] and experiments for survival under abiotic or biotic conditions, genes important for *A*. *baumannii* viability in bacteriological medium, colonization in murine hosts or human ascites have been identified [15, 16].

A number of classical methods, including transposon mutagenesis, gene deletion by recombination-mediated genetic engineering [17] or by suicide vectors such as those derived from the R6K plasmid [18] have been successfully used in genetic manipulation of *A*. *baumannii* [12]. For genetic complementation and ectopic gene expression in this bacterium, scientists currently rely on only shuttle plasmids derived from the cryptic plasmid pWH1277 originally isolated from *A*. *calcoaceticus* [19]. Replication of these plasmids in *Escherichia coli* was achieved by incorporating such origin of replication as the ColE1 [20]. These plasmids have been proven highly instrumental in assessing the role of its genes in various experimental settings. Recently, Visca and colleagues reported pVRL1 and pVRL2, two shuttle plasmids that are based on pWH1277 and the ColE1 origin of replication [21]. These plasmids and their derivatives carry not only selection markers that allow their use in MDR strains, but also a toxin-antitoxin module to facilitate their maintenance in *A*. *baumannii* [21]. Furthermore, the authors demonstrate that the arabinose inducible promoter P_BAD_ [22] can be used to finely control gene expression in *A*. *baumannii* [21], which clearly will be highly useful in the study of this pathogen.

Members of the IncQ plasmids originally identified in and *Escherichia coli* are known for their broad host range, relatively small size and the ability to be recognized by diverse type IV secretion systems [23], including some that are dedicated for bacterial virulence by transporting effector proteins such as the VirB system of *Agrobacterium tumefaciens* [24] and the Dot/Icm system of *Legionella pneumophila* [25]. Simultaneous expression of more than one gene in a given bacterial strain is required for such experiments as complementation of mutations in two different genes, interactions of two proteins and the study of gene regulation with plasmid-borne reporters and regulatory proteins. To accommodate such needs, we attempted to identify cloning vehicles different from pWH1277 and its derivatives by testing a number of well-established plasmids from different Inc groups. We found that plasmids derived from RSF1010 of the IncQ group [26] can stably replicate in *A*. *baumannii*. To increase its usefulness, we constructed two vectors, pJL01 and pJL02 from the IncQ plasmid pDSK519 [27] that allow arabinose- and IPTG-inducible gene expression respectively in *A*. *baumannii*. To facilitate detection of the protein of interest by immunoblotting, we have added a Flag tag [28] upstream of the multicloning region. Two other plasmids, pJL03 and pJL04 with similar inducible promoters and epitope tags, were also constructed from pVRL1 [21]. Furthermore, we showed that these plasmids allow regulated expression of proteins in *A*. *baumannii*, and that pJL01 and pJL04 permit simultaneous expression of proteins controlled by their respective promoter and the corresponding inducer.

## Materials and methods

### Bacterial strains, medium, bacterial transformation and conjugation

The bacterial strains used in this study are listed in **Table 1**. *A*. *baumannii* and *E*. *coli* were grown in Luria-Bertani broth at 37°C or in the Vogel-Bonner minimal medium with succinate (VBS) as the sole carbon source [29]. When necessary, antibiotics were used as follows: For *E*. *coli*, ampicillin, 100 μg/mL, gentamicin 10 μg/mL, and kanamycin, 30 μg/mL. For *A*. *baumannii*, kanamycin, 30 μg/mL, gentamicin, 30 μg/mL; streptomycin, 100 μg/mL.

**Table 1:** Bacterial strains and plasmids used in this study.

Strains or plasmids	Relevant features	Source
*A*. *baumannii*		
ATCC 17978	Type strain	[15]
ATCC 17978Strp^R^	Strep^R^, a derivative of ATCC 17978 resistant to streptomycin	This study
17978Δ*hisD*	Deletion mutant of the histidinol dehydrogenase gene (*hisD*, A1S_0687)	This study
*E*. *coli*		
DH5	*E*. *coli* strain for molecular cloning	[50]
BL21(DE3)	*E*. *coli* strain for protein expression from the T7 RNA polymerase	[51]
S17-1	*E*. *coli* strain for biparental conjugation, *recA pro hsd*R, RP4-Tc::Mu-Km::*Tn7*	[31]
MT607(pRK600)	Cm^R,^ *E*. *coli* helper strain for triparental conjugation,	[32]
Plasmids		Source
pDSK519	Km^R^, an IncQ plasmid derived from RSF1010	[27]
pBAD22	Amp^R^, A ColE1 plasmid carrying the P_BAD_ promoter and the *rrnB* T1T2 transcriptional terminators	[22]
pBAD22Bam-1	A derivative of pBAD22 lacking a BamHI site in the end of the P_BAD_ promoter	This study
peGFPC1	Km^R^, source for the *gfp* gene	Clontech
pJY100	Gm^R^, a synthetic plasmid harboring the *lacI*^Q^ gene, the P_TAC_ promoter and 5 copies of the *lac* operator	This study
pVRL1	Gm^R^, shuttle plasmid for *A*. *baumannii*	[21]
pVRL1StuI	Gm^R^, a derivative of pVRL1 with a *Stu*I restriction site	This study
pJL01	Km^R^, a pDSK519-based plasmid harboring P_BAD_	This study
pJL02	Km^R^, a pDSK519-based plasmid harboring P_TAC_	This study
pJL03	Gm^R^, a pVRL1-based plasmid harboring P_BAD_	This study
pJL04	Gm^R^, a pVRL1-based plasmid harboring P_TAC_	This study
pSR47s	Km^R^, *sacB*, a R6K plasmid for gene deletion	[35]
pSR47sΔhisD	Km^R^, a pSR47s derivative for deleting *hisD* (A1S_0687) in *A*. *baumannii*	This study
pET28a::ICDH	Km^R^, a plasmid for the production of the ICDH protein of *A*. *baumannii* in *E*. *coli*	This study
pJL01::LacZ	Km^R^, the *E*. *coli lacZ* gene cloned in pJL01	This study
pJL02::LacZ	Km^R^, the *E*. *coli lacZ* gene cloned in pJL02	This study
pJL03::LacZ	Gm^R^, the *E*. *coli lacZ* gene cloned in pJL03	This study
pJL04::LacZ	Gm^R^, the *E*. *coli lacZ* gene cloned in pJL04	This study
pJL01::HisD	Km^R^, the *A*. *baumannii hisD* gene cloned in pJL01	This study
pJL02::HisD	Km^R^, the *A*. *baumannii hisD* gene cloned in pJL02	This study
pJL03::HisD	Gm^R^, the *A*. *baumannii hisD* gene cloned in pJL03	This study
pJL04::HisD	Gm^R^, the *A*. *baumannii hisD* gene cloned in pJL04	This study
pJL01::eGFP	Km^R^, the enhanced *gfp* gene cloned in pJL01	This study

Note: Cm, chloramphenicol; Km, kanamycin, Gm, gentamicin; Strep, streptomycin.

Electrocompetent cells of A. *baumannii* were prepared as described previously with minor modifications [30]. Briefly, bacteria grown in LB broth for 18 h were diluted 1:100 into 50 mL LB and the cells were grown in a shaker at 37°C for 24 h. Cells were harvested by centrifugation at 3,000x*g* for 15 min, washed twice with 25 mL of 10% glycerol at room temperature. Cells suspended in 1.5 mL of 10% glycerol were aliquoted in 50 μL and stored at 80°C. Electroporation was performed by placing a mixture made from mixing 300 ng (1 μL) of plasmid DNA with 50 μL of competent cells to a 0.2-cm electroporation cuvette in a Gene Pulser (Bio-Rad) set at 2.5 kV/cm, 200 Ω, 25 μF. 1 ml of prewarmed LB broth was added immediately to transformed samples and the cells were allowed to recover by incubation at 37°C for 1 h.

For bacterial conjugation, 100 μl of saturated cells of donor strains, recipient strains and if necessary helper strain were mixed in a test tubes, and the cells collected by centrifugation for 1 min at 500x*g*, cells were washed with 50 μL fresh LB broth and the mixed cells in 50 μL medium were spotted onto nitrocellulose membranes were placed on the surface of LB agar. For bi-parental conjugation, the donor strains are derived from S17-1 [31] and for tri-parental mating, the *E*. *coli* helper strain MT607 which carries the helper plasmid pRK600 [32] were used. After incubation for 4 h at 37°C, cells washed off the membranes with PBS were diluted and plated onto selective medium. The colony forming units were counted. The number of donor cells used in the conjugation was determined by serial dilution of the cultures used for the experiments. Conjugation efficiency was calculated by dividing the number of transconjugants by the number of donor cells in each experiment.

To test plasmid stability, the testing bacterial strains were grown to saturation in LB broth with appropriate antibiotic selection and the cultures were diluted in fresh medium at 1:100. After culturing for 24 h in a shaker, the bacterial cells were serially diluted and plated onto LB plates without antibiotics and with the appropriate antibiotics, respectively. The number of CFU was determined. For the 48 h assay, cultures of the 24 h experiments were similarly further diluted into fresh medium, grown for an additional 24 h prior to determining the number of cells that maintained the ability to confer resistance to the antibiotics.

### DNA manipulation, vector construction and plasmid copy number determination

Oligonucleotides used in this study were listed in **S1 Table**. Bacterial genomic DNA was isolated using a TIANGEN DNA minikit (TIANGEN), and plasmid DNA was purified using TIANGEN Plasmid Extraction Kits. PCR reactions were performed with the high fidelity *Pfu* DNA polymerase. Unless otherwise noted, restriction enzymes and T4 DNA ligase were purchased from NEB.

**Fig 1:**
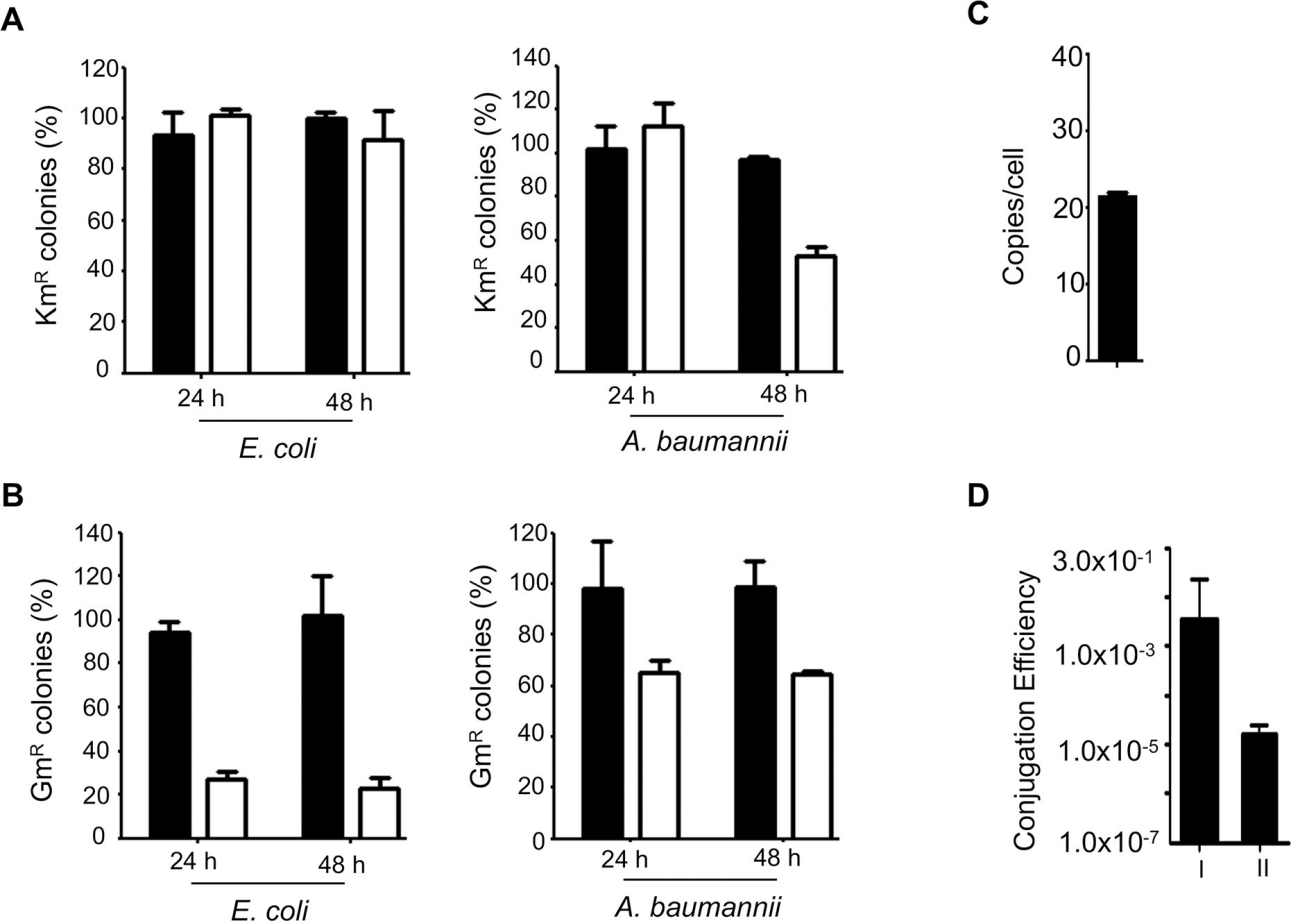
The stability of the IncQ plasmid pDSK519 in *E*. *coli* and *A*. *baumannii*. **A-B**. Saturated cultures *E*. *coli* strain DH5a (left) and *A*. *baumannii* strain ATCC 17978 (right) harboring pDSK519 (A) or pVRL1 (B) were diluted 1:100 in fresh LB broth with (solid bars) or without (open bars) antibiotics and grown for 24 h and cells retaining the plasmid were determined. Data for the 48 h time point were obtained from further diluted cultures grown for an additional 24 h in LB broth without antibiotics. **C**. Plasmid copy number of pJL02 in *A*. *baumannii*. Plasmid copy number per cell was determined as described in Materials and Methods, and the results shown are average from three independent experiments each done in triplicate. Data shown were means ± SEM. **D**. Introduction of the IncQ plasmid pDSK519 into *A*. *baumannii* by conjugation. Cells from cultures of the donor, helper and the recipient strain (I) mixed at a 1:1:1 ratio were placed on nitrocellulose membranes for 4 h on LB plates. Transconjugants appeared on selective medium containing streptomycin and kanamycin were enumerated. The number of donor cells used for the experiments was similarly determined. For bi-parental conjugation, the donor strain and the recipient were mixt at 1:1 (II). In all cases, samples were done in triplicate and similar results were obtained in three independent experiments. Data shown were means ± SEM.

To construct pJL01, a fragment containing *araC* and the P_BAD_ promoter was amplified from pBAD22 [22]. Elements such as the Flag tag, the SD sequence and restriction sites shown in Fig 1A were added to the 5’ end of the relevant oligonucleotide. The PCR product was digested with *Pci*I and *Pst*I and inserted into similarly digested pDSK519. To construct pJL02 in which gene expression is driven by the P_TAC_ promoter, a DNA fragment with sequences for such elements as the promoter, Flag tag, the *lac* operators were synthesized and ligated to a DNA fragment containing *lacI*Q driven by the *rpoD* (A1S_2706) promoter of *A*. *baumannii* also obtained by synthesis to give pJY100. The entire controlling region was then amplified by PCR and inserted into pDSK519 that had been digested with *Pci*I and *Kpn*I. Note that in both pJL01 and pJL02, the start codon ATG is part of the recognition sequence (-CCATGG-) for the restriction enzyme *Nco*I, which is unique in both plasmids. This *Nco*I site can be used to insert DNA fragments carrying desirable epitope tags different from Flag.

**Fig 2:**
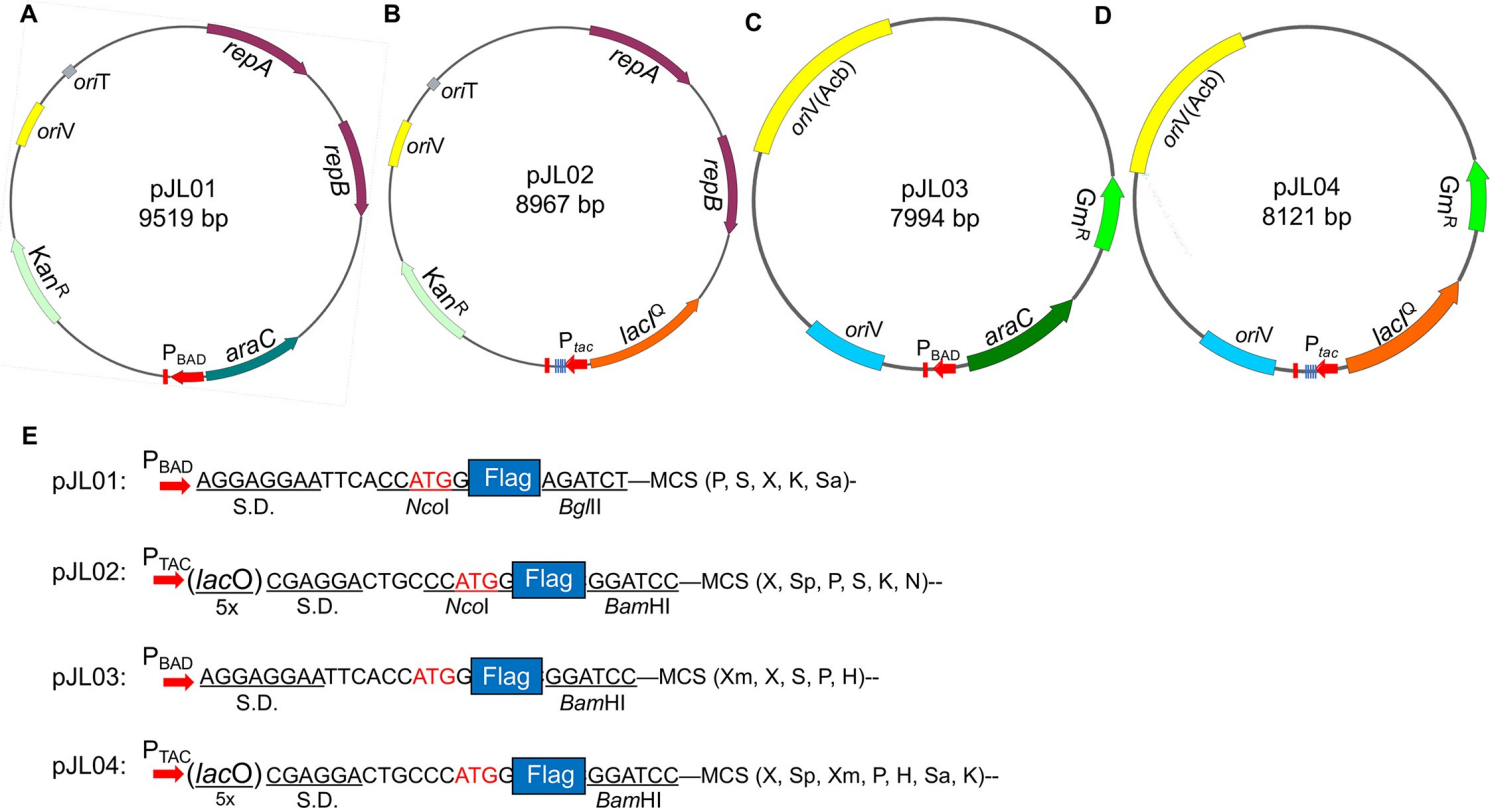
Diagrams of the four plasmids constructed in this study. **A-D.** Important features of pJL01 (A), pJL02 (B), pJL03 (C) and pJL04 (D) are indicated on plasmid maps in distinct colors. The promoters P_BAD_ and P_TAC_ are indicated by red arrows at the bottom of each map. In each case, the red bar downstream of the promoter represents the T1T2 transcriptional terminators of the *rrnB* ribosomal gene. The five vertical bars downstream of the P_TAC_ promoter in pJL02 and pJL04 represent the five copies of the *lac* operator. Other features such as antibiotic resistance genes and origins of replication are labeled on the maps. All maps were generated by the SnapGene software (GSL Biotech). **E.** The configuration of the elements for protein translation in the four plasmids. In each case, the sequence of the SD element, the space between the SD element and the translational start codon, the position of the Flag tag and the first restriction enzyme suitable for making Flag tagged fusions are indicated. Abbreviations for shown restriction enzymes: H, *Hind*III; K, *Kpn*I; N, *Not*I; P, *Pst*I; S, *Sac*I; Sa, *Sal*I; Sp, *Spe*I; X, *Xba*I; Xm, *Xma*I. Note the *Nco*I site immediately upstream of the sequence coding for the Flag tag in pJL01 and pJL02 that can be used to insert DNA fragments carrying alternative epitope tags. The information of additional restriction sites is available in the complete sequences of the plasmids.

To construct the pVRL1-based pJL03 and pJL04, we first introduced an A2209T mutation located downstream of the MCS region of pVRL1 [21], which created a *Stu*I site to give pVRL1StuI. Next, we introduced a C1224G mutation in pBAD22 [22] to eliminate the *Bam*HI restriction site in this location to give pBAD22Bam-1. The P_BAD_ promoter region of pBAD22Bam-1 was amplified with another primer pair and inserted into pVRL1 as an *Aat*II*/Stu*I fragment to give pVRL1PBAD. Then a DNA fragment obtained by annealing oligonucleotides JL1011 and JL1012 was inserted into *Nco*I/*Bam*HI digested pVRL1PBAD. These manipulations produced pJL03, which contains the P_BAD_ promoter that is configured with the Flag tag and other elements (**Fig 2**). Finally, a DNA fragment containing *lacI*^Q^, the P_TAC_ promoter and other elements was amplified from pJY100 and cloned into pVRL1 as an *Aat*II/*Stu*I fragment to give pJL04.

The coding region of the *lacZ* gene amplified from genomic DNA of *E*. *coli* strain S17-1 [31], digested with *Bgl*II and *Sal*I, and inserted into similarly digested pJL01, pJL02, pJL03 and pJL04, respectively. The coding sequence for enhanced GFP was amplified from pEGFPC1 (Clontech), digested with *BamH*I/*Sal*I and inserted into similarly digested pJL01 to give pJL01::GFP. All constructs were verified by sequencing analysis.

Plasmid copy number was determined by real-time quantitative qPCR (RT-qPCR) by an experimental procedure described earlier [33]. Briefly, serially diluted DNA solution of pJL02 of known concentrations was used as template to generate a standard curve by RT-qPCR. Cultures of *A*. *baumannii* were diluted to approximately 1x10^7^ cfu/mL, boiled for 5 min and used for RT-qPCR with primers Km_Fwd and Km_Rev (**S1 Table**). In each case, a 20-μl reaction contains: 10 μl SYBR (Roche), 0.5 μl forward primer, 0.5 μl reverse primer, template 1.0 μl and 8 μl H_2_O. Reactions were performed using a StepOnePlus real-time PCR system (ThermoFisher). Cell number was determined by plating serially diluted cultures onto LB plates.

### *A*. *baumannii* mutant construction and complementation

The gene coding for the histidinol dehydrogenase (*hisD*, A1S_0687) [34] was deleted from the genome of strain 17978 as described earlier [13]. Briefly, we first isolated a streptomycin resistant mutant of strain 17978 by plating approximately 3x10^8^ cells on LB plates containing 100 μg/mL streptomycin, and one colony appeared on the selection medium was purified and used for subsequent experiments. To construct the deletion plasmid, 0.8 kilobases DNA fragments upstream and downstream of A1S_0687 were amplified by PCR and ligated into pSR47s, a R6K-based suicide plasmid for gene deletion [35]. The primers were designed so that the first and last 15 amino acids of the deleted gene will remain in the mutant. The plasmid pSRΔHisD was introduced into strain ATCC17978 by triparental mating and the transconjugants resistant to kanamycin and streptomycin were selected, purified. Colonies appeared on plates containing 5% sucrose were screened for the deletion A1S_0687 by PCR reactions with appropriate primer pairs.

The *hisD* gene was inserted into each of the four plasmids and the resulting strains were used to examine complementation as follows: Cells for each strain grown to saturation were diluted 1000-fold in PBS and 7-μl of the cell suspension was spotted onto VBS solid medium supplemented with different concentrations of arabinose or IPTG. The growth of the cells was examined after 16 h incubation at 37°C.

### β-galactosidase activity assay

Overnight cultures of the testing bacterial strains were diluted in fresh LB broth at 1:20, and cultures grown to OD_600_ of 0.6–0.8 were split into 2-mL subcultures into which arabinose or IPTG was added at the indicated concentrations. After incubation in a shaker for an additional 4 h, bacterial cells were collected to measure b-galactosidase activity as described earlier [36]. 100-μl cells from induced cultures were collected for enzymatic assay with ONPG as the substrate following the standard protocol [36]. All samples were performed in triplicate and the activity was expressed as Miller unit.

### Antibodies, protein expression induction and immunoblotting

The antibody for the Flag tag was purchased from Sigma (Sigma, Cat# F1804) and was used at 1: 3000 for immunoblotting. To prepare the antibodies specific for the isocitrate dehydrogenase (ICDH)(A1S_2475), the coding sequence of this gene was inserted into pET28a (Novagen) and the resulting plasmid was transformed into *E*. *coli* strain BL21(DE3) for protein expression. Briefly, saturated bacterial cultures were diluted into fresh LB with the appropriate antibiotics at 1:50. When OD_600_ of the cultures reached 0.6, IPTG was added to a final concentration of 0.2 mM. The cultures were further incubated in a shaker (180 rpm/min) at 16°C for 16 h. Bacterial cells harvested by centrifugation were suspended in 30 mL lysis buffer (50 mM NaH_2_PO_4_, 300 mM NaCl, 10 mM imidazole, pH 8.0) and lysed using a JN-Mini Low Temperature Ultrahigh Pressure Continuous Flow Cell Cracker (JNBIO, Guangzhou, China). The soluble fraction containing the protein of interest was obtained by centrifugation at 12,000x*g* for 20 min and was mixed with Ni^2+^-NTA beads (Qiagen) for 1.5 h by rotation at 4°C. The beads were loaded onto a column and unbound proteins were removed by washing with 3 times of column volumes of washing buffer (50 mM NaH_2_PO4, 300 mM NaCl, 20 mM imidazole, pH 8.0). Bound His_6_-tagged proteins were eluted with 5 mL of elution buffer (50 mM NaH_2_PO4, 300 mM NaCl, 250 mM imidazole, pH 8.0). The purified proteins were dialyzed in a buffer containing 25 mM Tris-HCl (pH7.5), 150 mM NaCl and 10% (v/v) glycerol. Protein concentration was determined using the Bradford assay with BSA as the standard. Antibodies were raised in rabbits by standard protocols (AbMax Biotechnology Co., LTD, Beijing, China). The antisera were used at 1:2000 in immunoblotting.

Cultures used to detect the expression of proteins in *A*. *baumannii* were grown and induced as described for the β-galactosidase assay. Cells equivalent to 1.0 OD_600_ (approximately 1.0x10^9^ cells) were lysed with SDS sample buffer by boiling for 5 min. Total proteins in the soluble fraction were separated by SDS-PAGE and transferred onto nitrocellulose membranes for immunoblotting as described [37]. Proteins were detected by incubation with appropriate primary antibodies and IRDye-conjugated secondary antibodies (Li-Cor). The signals were detected using an Odyssey® CLx Imaging System (Li-Cor).

### Data quantitation, statistical analyses

Student’s *t*-test was used to compare the mean levels between two groups each with at least three independent samples.

### Accession numbers

The GenBank accession numbers for full-length sequences of pJL01, pJL02, pJL03 and pJL04 are MT370811, MT370812, MT370813 and MT370814, respectively.

## Results

### IncQ plasmids can stably replicate in *A*. *baumannii*

To identify additional cloning vehicles capable of replicating in *A*. *baumannii* autonomously, we examined a number of broad-host-range plasmids for their ability to transform this bacterium, including pRK415K of the IncP group [38], pDSK519 of the IncQ group [27] derived from RSF1010 [39] and pBBR1MCS2, which is of an unknown Inc group but can coexist with plasmids from many different Inc groups [40]. Although numerous plasmids of these Inc groups have been constructed, we chose these three for our initial tests because each of them harbors a kanamycin resistance gene, which makes the screening more straightforward for the commonly used *A*. *baumannii* strains such as ATCC_17978, whose genome had been sequenced and well-annotated [34]. When DNA of each of these plasmids was introduced into *A*. *baumannii* by electroporation, transformants resistant to kanamycin only appeared in samples receiving pDSK519, in which an average of approximately 2.3x10^3^ colonies were obtained in transformation with 0.2 μg plasmid DNA, suggesting that this vehicle can autonomously replicate in this bacterium.

We further investigated the replication of pDSK519 in *A*. *baumannii* by examining its stability in *A*. *baumannii* and *E*. *coli*. We used a continuous culturing method to determine the stability of the plasmid. In *E*. *coli*, almost all of the cells still harbored pDSK519 after 24-h growth of 1000-fold diluted cultures in medium without kanamycin, the selection antibiotics. Approximately 90% of the cells appeared to retain the plasmid after another round of dilution and an additional 24-h culturing (**Fig 1A, left panel**). In *A*. *baumannii*, the plasmid retaining rates are similar to those of *E*. *coli* in the first 24 h, with virtually all bacteria harboring the plasmid. The rates dropped to 50% after another 24-h growth of further diluted cultures in LB broth without selection pressure from antibiotics (**Fig 1A, right panel**). As expected, all cells in cultures derived from medium containing kanamycin were resistant to the antibiotic (**Fig 1A, solid bars)**.

As a comparison we also determined the stability of pVRL1, a previously described plasmid that contains origins of replication for *E*. *coli* and *A*. *baumannii*, respectively [21]. Under our experimental conditions, in *E*. *coli*, approximately 28% of the cells retained the plasmid after 24-h growth in LB broth without gentamicin selection, and the rates slightly dropped to about 22% after an additional 24 h incubation of further diluted cells (**Fig 1B, left panel**). In contrast, the stability of pVRL1 in *A*. *baumannii* is considerably higher, with about 65% of the cells retaining the plasmid after 24-h growth in the absence of gentamicin, the rate did not further decrease after an additional 1-day incubation of further diluted cultures (**Fig 1B, right panel**). All cells in the control cultures containing gentamicin were able to form colonies on plates seeded with the antibiotic (**Fig 1B, solid bars).**

Using a qPCR-based method [33], we determined the copy number of pDSK519 in *A*. *baumannii* to be approximately 20 copies/cell (**Fig 1C)**, which is slightly higher than the 12 copies/cell found in *E*. *coli* [41]. This copy number is considerably lower than that of pVRL1, which had been determined to be about 58 [21].

Plasmids of the IncQ group such as pDSK519 derived from RSF1010 are mobilizable by the conjugal transfer system of members of the IncP group [27]. We thus examined the effectiveness of conjugation in introducing pDSK519 into *A*. *baumannii*. When a mixture of cells containing strain DH5a(pDSK519), *A*. *baumannii* strain ATCC_17978 and the *E*. *coli* helper strain MT607 that harbors pRK600 ColE1 replicon with RK2 *tra* genes [32], at a 1:1:1 ratio was incubated on LB plates for 4 h, we observed a conjugation efficiency of approximately 2.3x10^-1^ per donor cell on selective medium containing streptomycin and kanamycin. The transfer efficiency was about 1.1x10^-1^ per donor cell in bi-parental conjugation using the *E*. *coli* strain S17-1 [31] as the donor (**Fig 1D**). Taken together, these results indicate that plasmids of the IncQ group can autonomously replicate in *A*. *baumannii* and that the commonly used conjugation systems can be used to transfer these plasmids from *E*. *coli* into this pathogen.

### Construction of four plasmids suitable for controlled gene expression in *A*. *baumannii*

To increase the usefulness of IncQ plasmids in *A*. *baumannii* for experiments such as complementation of mutants with distinct phenotypes and expression of genes for other purposes, we constructed two pDSK519-derived vectors in which gene expression is driven by two promoters regulated by different mechanisms. The first plasmid pJL01 harbors the arabinose-inducible promoter [22], which has been shown to allow precise control of gene expression in a wide variety of bacteria [42], including *A*. *baumannii* [21]. To facilitate the detection of the gene products, we inserted a sequence element coding for the Flag tag preceded by an ATG start codon directly downstream of the optimized Shine-Dalgarno (SD) sequence (-AGGAGGAA-) [22] (**Fig 2**). Genes of interest can be inserted into the *Bgl*II site or other downstream restriction sites with primers that will make them in-frame with the upstream start codon. Following the multiple cloning sites (MCS) is the T1T2 transcriptional terminators from the *rrnB* gene coding for the *Escherichia coli* ribosomal RNA [43].

To expand the mechanism of gene expression regulation, we constructed pJL02 in which gene expression is achieved by the synthetic P_TAC_ promoter [44]. The following features were added to this vector for better inducibility: First, it contains five copies of the *lac* operator (*lac*O) sequence immediately downstream of the promoter. Second, the expression of the l*acI*^Q^ repressor that binds the *lac* operator is driven by the promoter of the sigma 70 gene (A1S_2706) of *A*. *baumannii* strain 17978. Third, similar to pJL01, a Flag tag sequence preceded by an optimized SD element was included to facilitate the expression and detection of the proteins. Finally, the T1T2 transcriptional terminators were added distal to the 3’ end of the MCS region (**Fig 2B**).

To allow simultaneous ectopic expression of more than one gene in *A*. *baumannii*, we constructed two additional plasmids based on pVRL1 [21]. Among these, pJL03 harbors the P_BAD_ promoter and elements for expression regulation and detection similar to those on pJL01 and pJL04 contains the P_TAC_ promoters and its accessories akin to those on pJL02 (**Fig 2C–2E**).

### Expression of genes by new plasmids in *A*. *baumannii*

**Fig 3:**
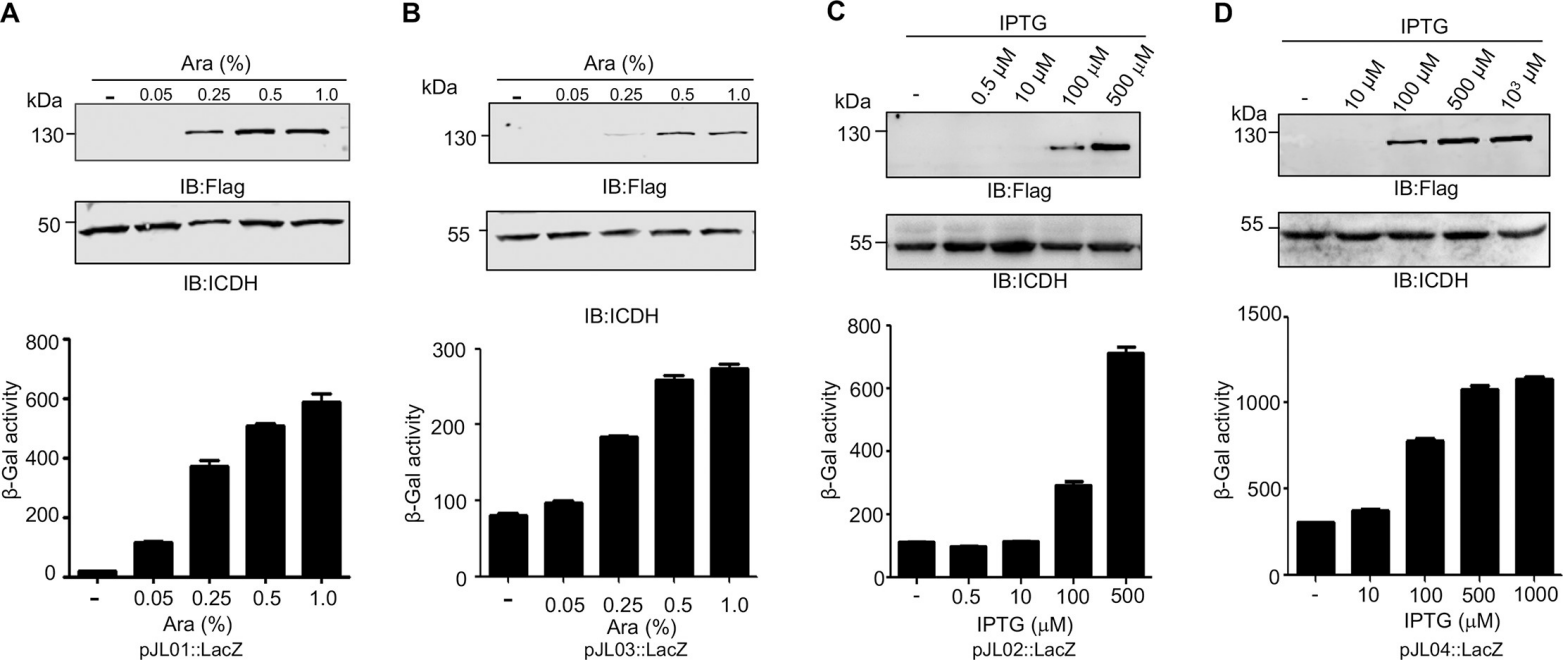
Controlled gene expression *A*. *baumannii* by the four plasmids. The coding sequence of the *lacZ* gene was inserted into each of the four vectors, pJL01 (**A**), pJL02 (**B**), pJL03 (**C**) and pJL04 (**D**). The resulting plasmids were introduced into *A*. *baumannii* strain ATCC 17978. Overnight cultures diluted at 1:20 into fresh medium grown to OD_600_ = 0.6 was induced by the arabinose or IPTG at the indicated concentrations for 4 h. Total proteins resolved by SDS-PAGE were detected by immunoblotting with the Flag-specific antibody (top panels). The metabolic enzyme isocitrate dehydrogenase was probed as loading controls (middle panels). Data shown were one representative from three independent experiments with similar results. Cells withdrawn from identical cultures were used to measure β-galactosidase activity. Data shown were means ± SEM and the experiments were done in triplicate.

We next examined the effectiveness of the four plasmids in protein production in *A*. *baumannii*, we inserted the coding sequence of the *lacZ* gene from *E*. *coli* into these plasmids and the resulting constructs were introduced into strain 17978. For pJL01 and pJL03 in which the expression of the inserted *lacZ* gene was driven by the P_BAD_ promoter, Flag-tagged LacZ was not detectable in samples not receiving the inducer arabinose (**Fig 3A and 3B**). In each case, induction with 0.05% arabinose for 2 h led to the production of considerable amounts of LacZ, and the activity of β-galactosidase in these samples increased correspondingly (**Fig 3A and 3B**). In these two strains, the expression of *lacZ* appears to plateau when the concentration of arabinose was increased to 0.5% as no further induction was observed when the inducer sugar was added to 1.0% (**Fig 3A and 3B**).

For vectors pJL02 and pJL04 that use the P_TAC_ promoter, in uninduced cultures or cultures receiving 10 μM IPTG, we did not detect Flag-LacZ by immunoblotting, and the enzymatic activity of LacZ among these cultures is comparable (**Fig 3C and 3D**), indicative of tight control of the promoter activity by LacI^Q^. In each case, the fusion protein became detectable in cultures receiving 100 μM IPTG although the expression level from pJL04 was detectably higher, and maximal expression was seen when IPTG was added at 500 μM. Furthermore, when the expression was induced by saturated amounts of IPTG, expression from pJL04 was considerably higher than that from pJL02 (**Fig 3C and 3D**), probably due to the differences in the copy number of these two plasmids.

**Fig 4:**
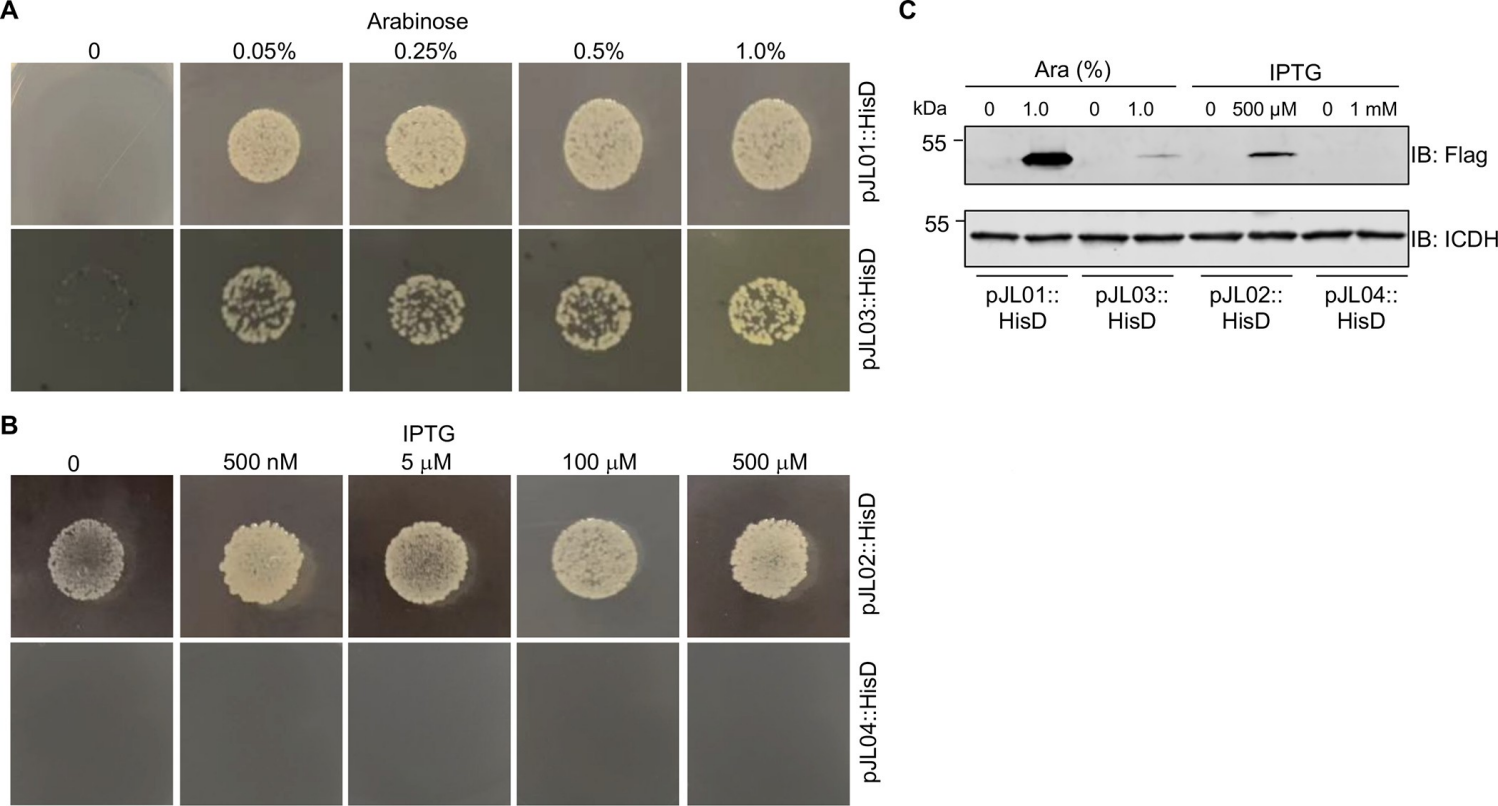
Complementation of an *A*. *baumannii* mutant by the four plasmids. **A-B**. The coding region of the histinol dehydrogenase gene *hisD* was inserted into each of the four vectors and the resulting plasmids, pJL01::HisD, pJL02::HisD, pJL03::HisD and pJL04::HisD were introduced into the *hisD* deletion mutant of *A*. *baumannii*. Cells from overnight cultures in LB broth supplemented with appropriate antibiotics were washed with PBS and diluted 1000-fold. Aliquots of 7-μL cells were spotted onto VBS medium and images were acquired after incubation at 37°C for 16 h. **C.** The expression of Flag-HisD in the complementation strains. Overnight cultures diluted in LB broth were grown to OD_600_ = 0.7 and arabinose and IPTG were added to the cultures at the indicated concentrations. After incubation at 37°C for 4 h, proteins solubilized by SDS sample buffer were separated by SDS-PAGE and Flag-HisD was detected by immunoblotting with the Flag-specific antibody. The metabolic enzyme isocitrate dehydrogenase (ICDH) was detected as a loading control.

To determine whether these plasmids can complement phenotypes caused by gene mutations in *A*. *baumannii*, we constructed a mutant of strain 17978 lacking the histidinol dehydrogenase gene (*hisD*, A1S_0687) [34] using the allele exchange method based on the suicide R6K plasmid pSR47s [35]. We then inserted the coding region of *hisD* into each of the four vectors and the resulting plasmids were introduced into the mutant by electroporation or conjugation. After washing 3 times with PBS, Cells grown in LB broth with appropriate antibiotic selection were diluted to approximately 3.0x10^6^ cells/mL and 7 μL of the cells were spotted onto Vogel-bonner minimal medium supplemented with succinate [29]. For the strain harboring pJL01::HisD, no growth of the bacteria was observed on plates without arabinose whereas the strain containing pJL03::HisD gave rise to a few small colonies on the edge of the spotting area on medium without the inducer (**Fig 4A**), suggesting that for this particular gene, pJL01 provides a tighter control than pJL03.

For the strain harboring pJL02::HisD, we observed robust growth of bacterial cells even on medium without IPTG (**Fig 4B. upper panels**). In contrast, the strain harboring pJL04::HisD, we did not observe complementation of the mutation even when IPTG was added to the medium as concentrations as high as 500 μM (**Fig 4B, lower panels**). Consistent with the no growth phenotype, we cannot detect the expression of the protein in this strain by immunoblotting with the Flag antibody in cultures induced with 1.0 mM IPTG (**Fig 4C**). We also observed less growth of strain Δ*hisD*(pJL03::HisD) than strain Δ*hisD*(pJL01::HisD), which is consistent with its lower protein levels detected by the Flag antibody (**Fig 4**). Taken together, these results indicate that, in *A*. *baumannii* the P_BAD_ promoter provides a better control in gene expression and that certain genes may not be properly expressed from some of these plasmids.

### Independent regulation of protein production from two plasmids in *A*. *baumannii*

**Fig 5:**
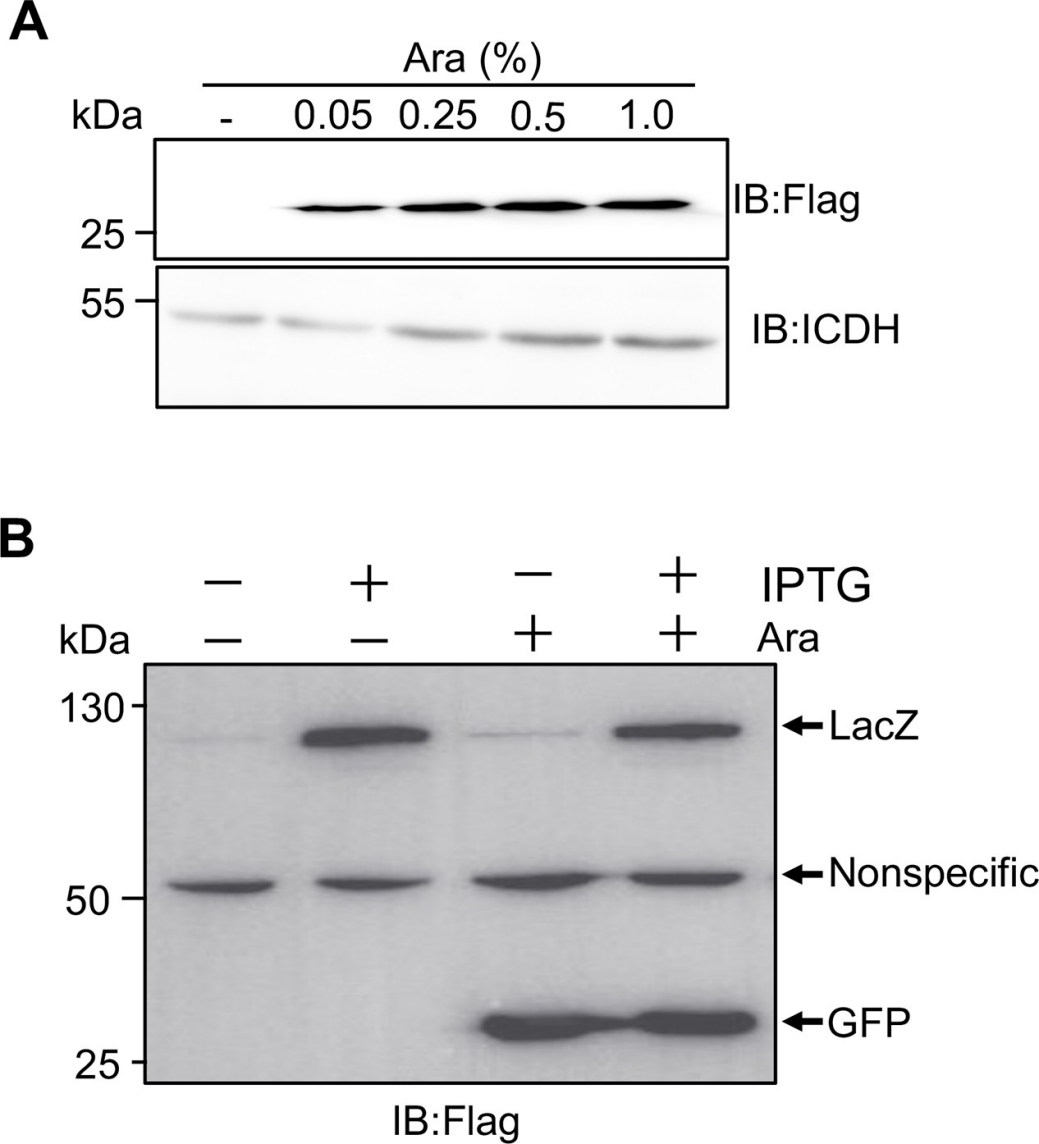
Independent protein expression by two plasmids co-existing in *A*. *baumannii*. **A**. The coding sequence of the green fluorescence protein (*gfp*) was inserted into pJL01 and the resulting plasmid was introduced into *A*. *baumannii* strain ATCC_17978. The expression of Flag-GFP was detected as described in Fig 3. **B**. Cultures of *A*. *baumannii* strain ATCC_17978(pJL04::LacZ, pJL01::GFP) were induced by arabinose, IPTG or both and the protein samples resolved by SDS-PAGE were detected by immunoblotting with the Flag-specific antibody. The signals of a band with a molecular weight of approximately 50 kDa detected nonspecifically by the antibody were used as a loading control. Results shown are one representative from at least three independent experiments with similar results.

One of the goals for the identification of plasmids that can coexist with derivatives of pWH1277 is to simultaneously express multiple genes in *A*. *baumannii*, we examined this possibility by inserting the green fluorescence gene *gfp* into pJL01. The resulting plasmid pJL01::GFP was then introduced into strain 17978 and strain 17978(pJL04::LacZ), respectively. The expression of Flag-GFP can be detected in strain 17978(pJL01::GFP) upon induction with arabinose at concentrations ranging from 0.05 to 1.0% (**Fig 5A**). In strain 17978(pJL01::GFP, pJL04::LacZ), induction with arabinose led to the expression of only Flag-GFP and induction with IPTG allowed the cells to solely produce Flag-LacZ detectable by the Flag antibody (**Fig 5B**). Importantly, in cultures receiving both arabinose and IPTG, Flag-GFP and Flag-LacZ were detected (**Fig 5B**). Thus, derivatives of the RSF1010 plasmid pDSK519 can replicate simultaneously with pWH1277-based vectors in *A*. *baumannii* to independently direct gene expression.

## Discussion

The development of vehicles suitable for gene cloning and expression has greatly facilitated our understanding of bacterial physiology, molecular biology and virulence. Infections caused by *A*. *baumannii* have increased at alarming rates, a problem that has been further compounded by the wide spread emergence of many MDR strains [3]. The high incidence of *A*. *baumannii* infection has recently attracted considerable research attention to this important opportunistic pathogen [6, 45]. The identification of plasmids that can coexist with the widely used vectors derived from pWH1277 [19] will expand the genetic toolbox for the research community working on *A*. *baumannii*.

It is not unexpected that vectors derived from the IncQ plasmid RSF1010 is able to autonomously replicate in *A*. *baumannii* because of their well-established wide host range [46]. Vectors from the IncQ group have been used in pathogens that appear more restrictive to plasmid replication, including *Legionella pneumophila* [47] and *Coxiella burnetii* [48].

Our results showed that LacZ can be expressed from pJL04, but the expression of the *hisD* gene from this vector is not successful (**Figs 3 and 4**). Although we cannot offer a sound explanation to this observation, this phenomenon is not completely unexpected. In experiments aiming to express and purify recombinant proteins from *E*. *coli* using standard expression vectors developed by various manufacturers, it is often to encounter occasions whereby genes inserted into some expression plasmids cannot be detectably expressed. One of the solutions to this problem is to use vectors that differ in their promoters, affinity tags or other features. Here, the problem is solved by the ability of the other three plasmids to express the *hisD* gene and complement the mutation (**Fig 4**). Another note is that strain Δ*hisD*(pJL02::HisD) grew on the histidine deficient medium without IPTG (**Fig 4B, lower panels**), suggesting that the control by LacI^Q^ in pJL02::HisD was not stringent and that the basal expression is sufficient to restore histidine biosynthesis in the mutant. One interesting but puzzling observation is that despite the plasmid copy number of pJL03 and pJL04 derived from pVRL1 is higher than that of RSF010-based pJL01 and pJL02, the latter plasmids expressed proteins at higher levels even when promoters and induction conditions were identical (**Figs 3 and 4).** We cannot offer a clear explanation for this phenomenon, but factors such as the configuration of the promoter relative to other elements on the plasmids may affect the promoter activity. Clearly, whereas plasmids that express at high levels are more suitable for experiments such as protein-protein interactions, those that express at lower levels may be more ideal for genetic complementation as the endogenous level of the protein of interest likely is low.

The availability of two different inducers and an epitope tag in these four vectors will facilitate the regulation and detection of gene expression in *A*. *baumannii*. As we have demonstrated, these vectors allowed simultaneous expression of two proteins in this pathogen (**Fig 5B**). To accommodate experiments such as the detection of protein-protein interactions, one can modify one or more of these vectors to carry other epitope tags such the hemagglutinin (HA) tag [49]. The presence of a unique *Nco*I site immediately upstream of the sequence coding for the Flag tag in pJL01 and pJL02 offers the flexibility for such modification (**Fig 2E**). One can design primers containing sequences for a desirable epitope tag such as HA and Myc and insert the PCR products into pJL01 or pJL02 digested with NcoI and an appropriate downstream restriction site. Clearly, such manipulation will allow the epitope tag to be added at either the amino or carboxyl end of the protein of interest. Similarly, the limitation of selection markers in their use in clinic MDR isolates, one can replace the gentamicin or kanamycin resistance gene in these plasmids with the zeocin resistance cassette [21] or other markers suitable for the strains of interest.

## Supporting information

S1 Table Oligonucleotides used in this study. (DOCX)

S1 File (PPTX)
