# Supplementary material for: A set of shuttle plasmids for gene expression in Acinetobacter baumannii
Source: PLoS One. 2021 Feb 10;16(2):e0246918. doi: 10.1371/journal.pone.0246918 (PMC7875395; doi:10.1371/journal.pone.0246918)
Supplement: S1 Table — (DOCX) [file pone.0246918.s001.docx]

**S1 Table. Oligonucleotides used in this study.**

| Primers | Sequence (Restriction enzyme sites are underlined) | Note |
| --- | --- | --- |
| pJL1001 | 5’-CTGACATGTCTATGCTACTCCGTCAAGC-3’ | pJL01 pBAD promoter_F |
| pJL1002 | 5’-CACCTGCAGAGATCTCTTGTCGTCATCGTC  TTTGTAGTCCATGGTGAATTCCTCCTGC-3’ | pJL01 pBAD  promoter _R |
| pJL1003 | 5’-ctgCTGGACGTCctatgctactccgtcaag-3' | pJL03 pBAD promoter_F |
| pJL1004 | 5’-ctgCTGAGGCCTgtttcacttctgagttcg-3' | pJL03 pBAD promoter _R |
| pJL1005 | 5’-CTGACATGTCGAGAAAAGGCCATCCGTC-3’ | pJL02-pTAC promoter-F |
| pJL1006 | 5’-CGGCCGCGGGTACCGTCGAC-3’ | pJL02-pTAC promoter-R |
| pJL1007 | 5'-ctgCTGGACGTCCGAGAAAAGGCCATCCGTC-3' | pJL04-pTAC promoter_F |
| pJL1008 | 5'-ctgCTGAGGCCTCGGCCGCGGGTACCGTCGAC-3' | pJL04-pTAC promotor-R |
| pJL1009 | 5'-AGCAAAAGGCCTGCAAAAGGCCA-3' | pVRL1^A2209G^_F |
| pJL1010 | 5'-CACATGTTCTTTCCTGCGTTATC-3' | pVRL1^A2209G^_R |
| pJL1011 | 5'-CATGGGCGATTACAAGGACGACGATGACAAGGGA  TCCGGGCCCGGGA-3' | pJL03-Flag_F |
| pJL1012 | 5'-GATCTCCCGGGCCCGGATCCCTTGTCATCGTCG  TCCTTGTAATCGCC-3' | pJL03-Flag_R |
| pJL1013 | 5'-CTGAGATCTACCATGATTACGGATTCAC-3’ | LacZ _F |
| pJL1014 | 5'-CTGGTCGACTTATTTTTGACACCAGACC-3’ | LacZ _R |
| pJL1015 | 5'-CTGGGATCCGTGAGCAAGGGCGAGGAG-3’ | eGFP_F |
| pJL1016 | 5'-CTGGTCGACTCACTTGTACAGCTCGTC-3’ | eGFP_R |
| pJL1017 | 5'-CTGGGATCCATGCGACGTTTATCGAC-3' | hisDA1S_0687_F |
| pJL1018 | 5'-CTGGTCGACTTATTGATAACGATAACGCG-3' | hisDA1S_0687_R |
| pJLl1019 | 5'-CTGGAGCTCCGAGTAAGTTAATGAATG-3' | 0687-KO_F1 |
| pJLl1020 | 5’-CTGGGATCCGGCAAAAACCTGTTTGAA-3’ | 0687-KO_R1 |
| pJLl1021 | 5’-CTGGGATCCAATCTCGATGCCCATGCA-3’ | 0687-KO-F2 |
| pJLl1022 | 5’-CTGGTCGACACAGTCTCAAGTGCTGCA-3’ | 0687-KO_R2 |
| Km_Fwd | 5’-GATGACGAGCGTAATGGCTG-3’ | Plasmid copy number determination |
| Km_Rev | 5’-TCGTCCAACATCAATACAACCTATTAATTT-3’ | Plasmid copy number determination |
