# Supplementary material for: A set of shuttle plasmids for gene expression in Acinetobacter baumannii
Source: PLoS One. 2021 Feb 10;16(2):e0246918. doi: 10.1371/journal.pone.0246918 (PMC7875395; doi:10.1371/journal.pone.0246918)
Supplement: S1 File — (PPTX) [file pone.0246918.s002.pptx]

## Slide 1
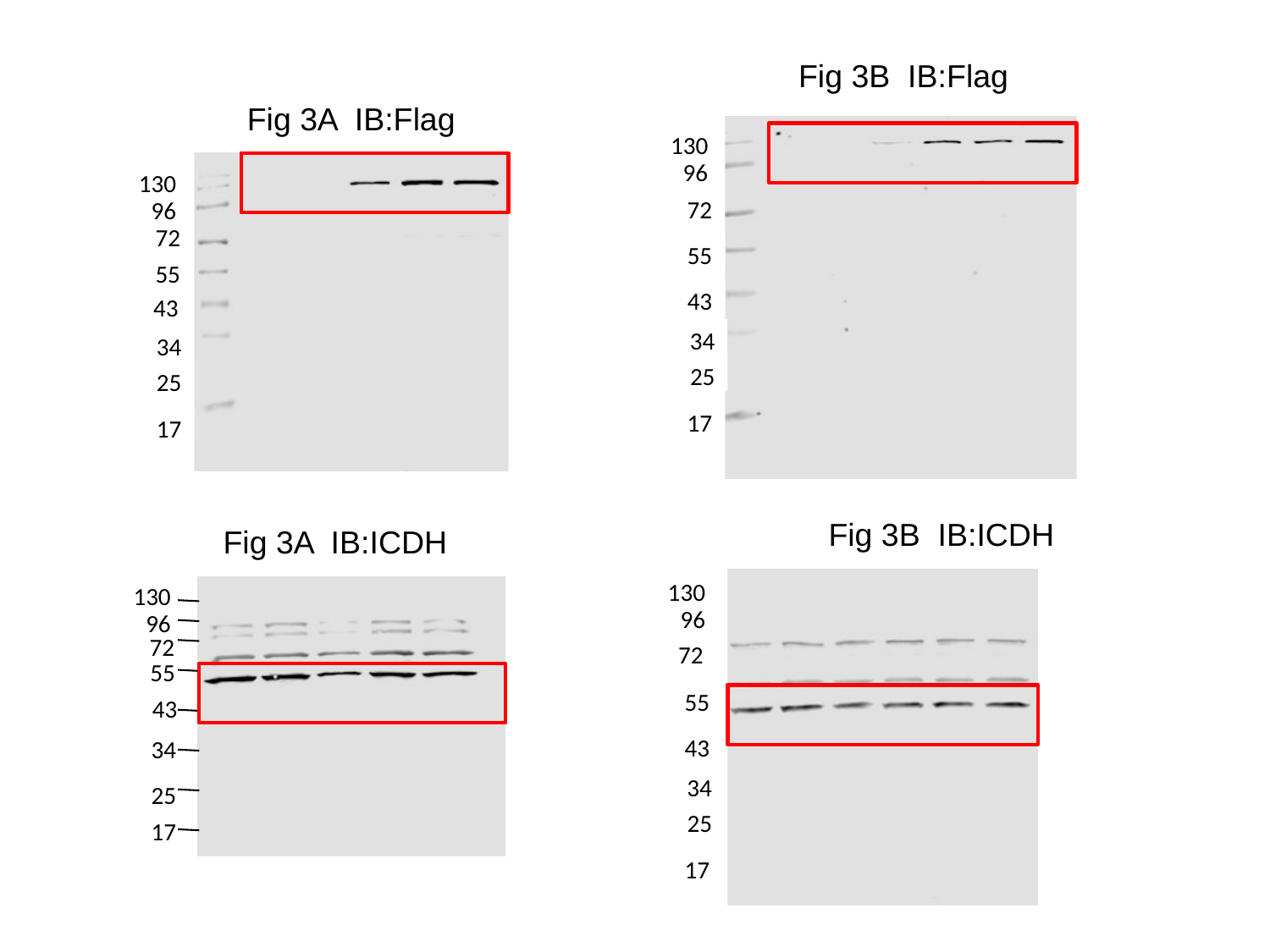

Fig 3B IB:Flag
Fig 3A IB:Flag
130
96
55
43
34
25
17
130
96
55
43
34
25
17
72
72
Fig 3B IB:ICDH
Fig 3A IB:ICDH
130
96
55
43
34
25
17
130
96
55
43
34
25
17
72
72

## Slide 2
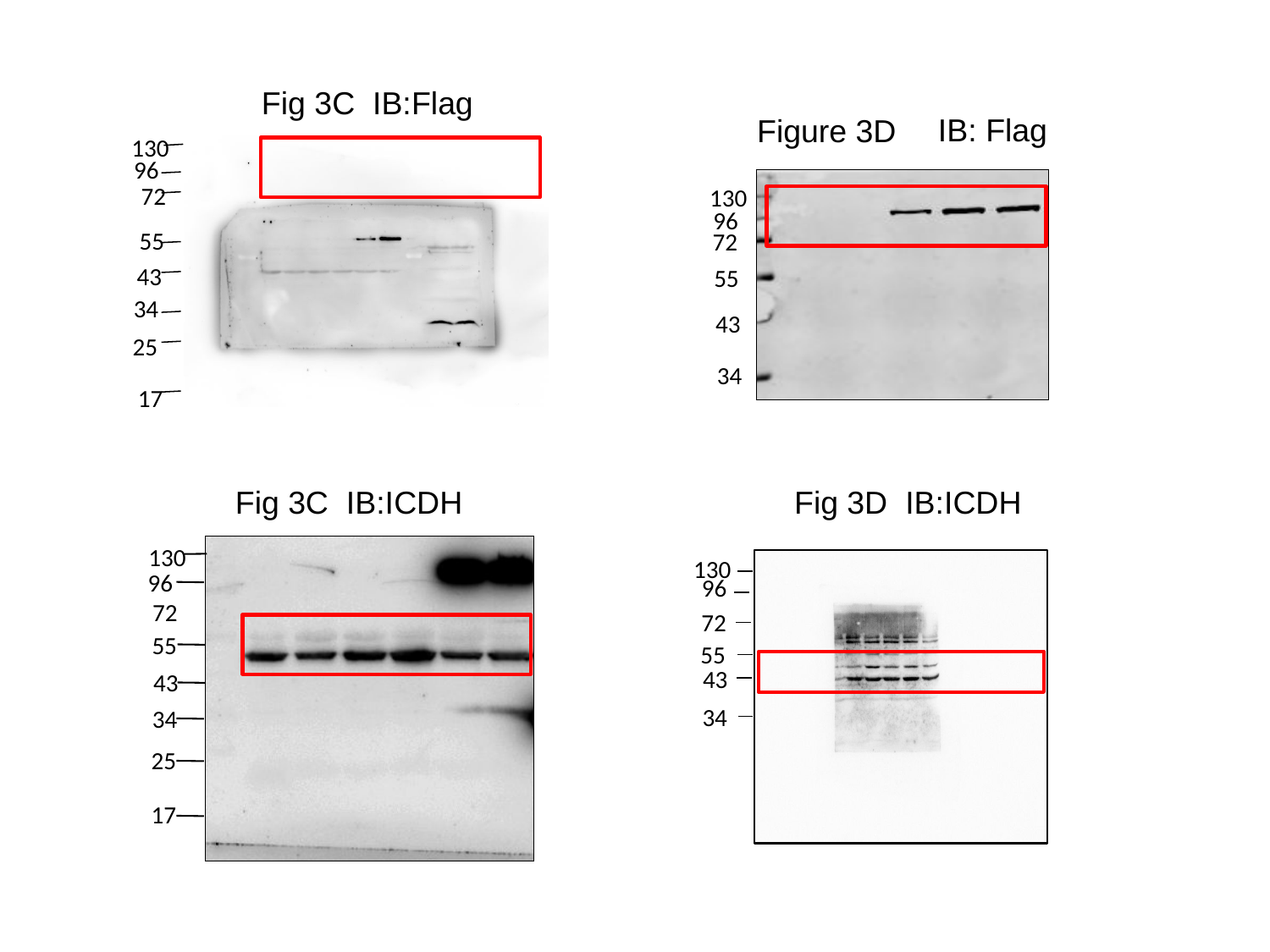

Fig 3C IB:Flag
IB: Flag
Figure 3D
 130
 96
 72
130
96
 55
72
 43
55
 34
43
 25
34
 17
Fig 3D IB:ICDH
Fig 3C IB:ICDH
130
96
55
43
34
25
17
72
130
96
72
55
43
34

## Slide 3
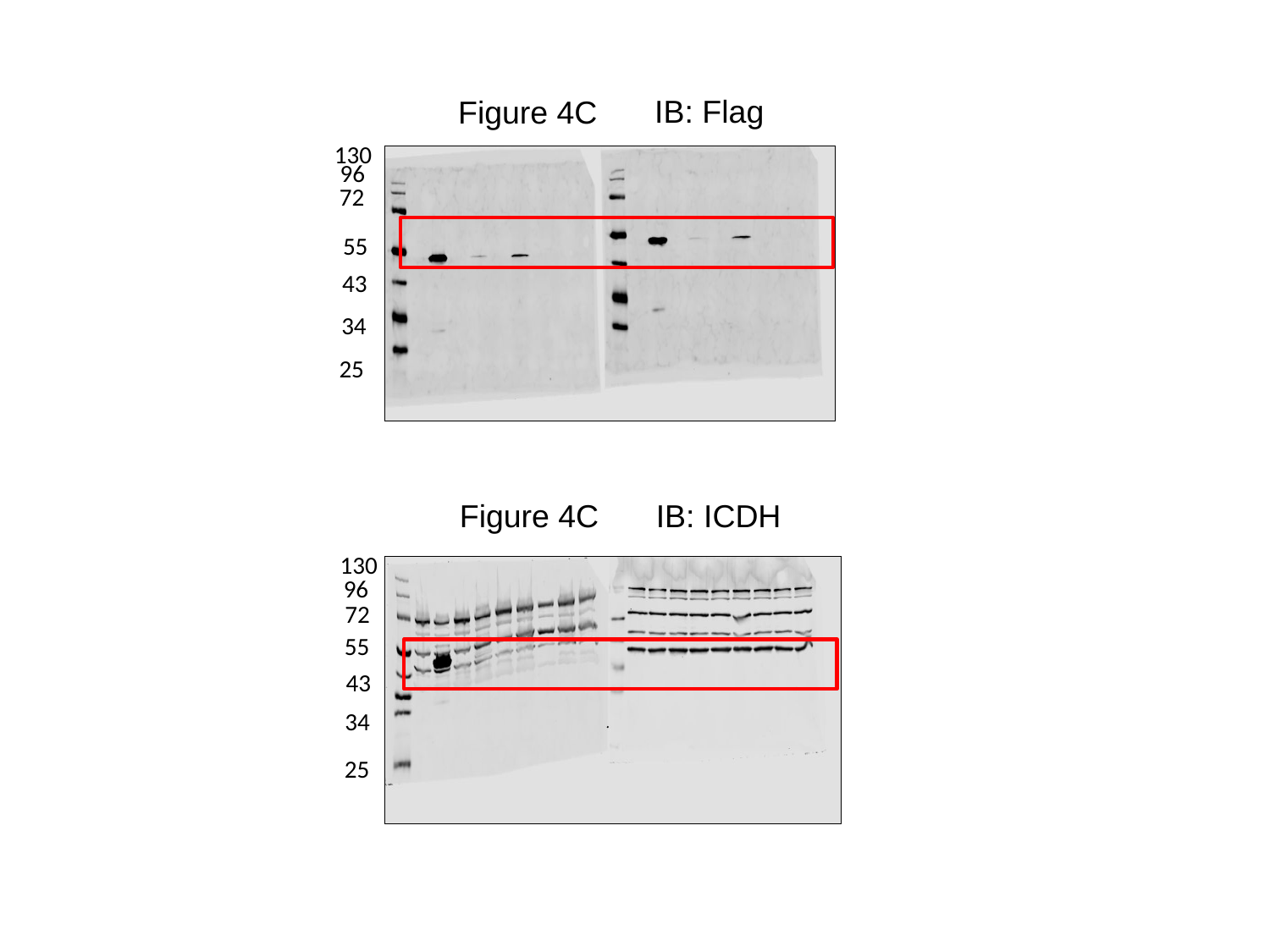

IB: Flag
Figure 4C
130
96
72
55
43
34
25
IB: ICDH
Figure 4C
130
96
72
55
43
34
25

## Slide 4
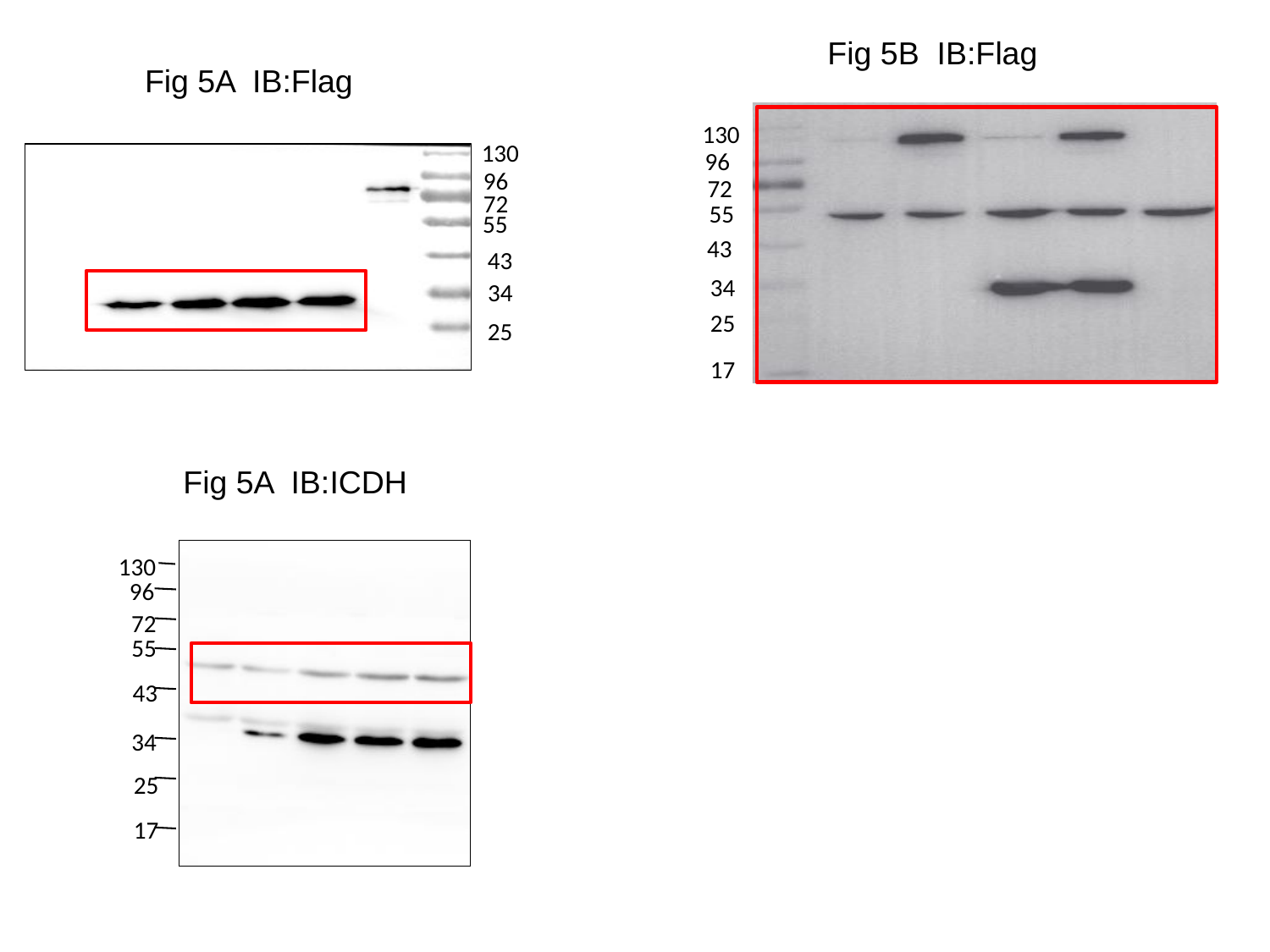

Fig 5B IB:Flag
Fig 5A IB:Flag
130
96
55
43
34
25
17
130
72
43
34
25
96
72
55
Fig 5A IB:ICDH
130
96
55
43
34
25
17
72
